# ReFernment: An R package for annotating RNA editing in plastid genomes

**DOI:** 10.1002/aps3.1216

**Published:** 2019-01-30

**Authors:** Tanner A. Robison, Paul G. Wolf

**Affiliations:** ^1^ Department of Biology Utah State University Logan Utah USA

**Keywords:** annotation, chloroplast, GenBank, genome, National Center for Biotechnology Information (NCBI), plastome, RNA editing

## Abstract

**Premise of the Study:**

In the absence of cDNA, the annotation of RNA editing in plastomes must be done manually, representing a significant time cost to those studying the organellar genomes of ferns and hornworts.

**Methods and Results:**

We developed an R package to automatically annotate apparent nonsense mutations in plastid genomes. The software successfully annotates such sites and results in no false positives for data with no sequencing or assembly errors.

**Conclusions:**

Compared to manual annotation, ReFernment offers greater speed and accuracy for annotating RNA editing sites. This software should be especially useful for researchers generating large numbers of plastome sequences for taxa with high levels of RNA editing.

The development of next‐generation sequencing has led to an explosion of available genome data, especially for plastid genomes (plastomes). These relatively small genomes are a major source of data for phylogenetic analyses. As of September 2018, more than 2700 plastome sequences from green plants have been published (https://www.ncbi.nlm.nih.gov/genomes/GenomesGroup.cgi?taxid=2759&opt=plastid) in public databases, which has in turn aided in the resolution of deep phylogenetic relationships across plant diversity (Ruhfel et al., 2014; Tonti‐Filippini et al., 2017; Gitzendanner et al., 2018). However, researchers assembling and annotating plastomes are often faced with the problem of RNA editing, whereby the sequence of the initial transcript is altered prior to translation. In some groups of plants, RNA editing can be high: up to 78% of protein‐coding genes in plastomes of ferns (Wolf et al., 2004) and hornworts (Kugita et al., 2003). Many of these RNA editing sites will alter the sequences of start codons, stop codons, or result in stop codons within the genomic coding sequence. The most common forms for RNA editing in plastomes are U‐to‐C or C‐to‐U editing (Kugita et al., 2003; Wolf et al., 2004). Whereas many of the automated annotation tools presently available are generally good at annotating plastid genes, none of them account for RNA editing (Wyman et al., 2004; Liu et al., 2012; McKain et al., 2017; Jung et al., 2018). This results in annotated genes that appear to be missing start codons or stop codons, or to contain numerous internal stops based on their nucleotide translations. Reasonably, issues like these make it difficult to get some plastome sequences approved for public databases such as GenBank.

Although RNA editing appears to occur at a lower rate in angiosperms than in other clades, 138 RNA editing sites were detected in the plastome of *Amborella* Baill. (Hein et al., 2016). Thus, the need to annotate RNA editing sites may not be restricted to a few seed‐free lineages. Tools are available to predict RNA editing sites, for example, PREPACT (Lenz and Knoop, 2013) and PREP suite (Mower, 2009). While these tools are powerful for predicting RNA editing sites in organelle genomes, they do not directly add to or alter an existing annotation file. Thus, in many cases, researchers manually add these annotations by examining each nonsense mutation and determining whether RNA editing would likely restore this site. This process, while necessary for admission to public repositories, is tedious and time‐consuming—especially considering that these edits to nonsense mutations occur in a highly predictable manner. Here, we attempt to solve this problem by introducing ReFernment, a simple R package that automatically annotates nonsense codons in DNA translations to account for RNA editing and provides conceptual translations for coding sequences. ReFernment is available at https://github.com/TARobison/ReFernment.

## METHODS AND RESULTS

ReFernment operates by refining existing annotations. Thus, the software uses an annotation generated by programs such as DOGMA, CpGAVAS, Verdant, or AGORA (Wyman et al., 2004; Liu et al., 2012; McKain et al., 2017; Jung et al., 2018) and adjusts these annotations to account for RNA editing. ReFernment requires both a GFF3 (no sequence) file and a GenBank flat file (including nucleotide sequence), and its basic operation is extremely simple. First, ReFernment checks the starting and final codons of each gene. In both cases, ReFernment initially checks whether the codon is a valid start or stop. If the codon is not valid, it checks whether an RNA editing event would result in the restoration of the codon to a valid start or stop (e.g., ACG to AUG). If the codon is not valid, even after checking for possible RNA editing, ReFernment checks whether nearby codons (within five codons) represent valid codons; if so, ReFernment changes the gene boundaries to start or stop at those valid sites. Next, ReFernment checks whether a gene has any internal stops, and if so, checks whether RNA editing would restore these nonsense mutations, adjusting the translation to account for this. ReFernment then edits the imputed GenBank flat file, adding conceptual translations and annotations indicating the sites where RNA editing occurred with ‘misc_feature’ flags, adding necessary RNA editing flags to the relevant genes, and providing a conceptual translation for each gene. Finally, ReFernment produces a five‐column feature table, formatted correctly for submission to GenBank, and a protein FASTA file with the conceptual translations for coding sequences where RNA editing has occurred.

ReFernment operates under the assumption that only U‐to‐C or C‐to‐U RNA editing is occurring in the plastome (Takenaka et al., 2013). Additionally, ReFernment assumes that all nonsense mutations are the result of RNA editing. Because most of the genes that reside within the plastome are vital to photosynthetic function, it is assumed that these genes will remain operational. There may be cases where internal stops, bad starts, or missing stops are actually the result of an uncorrected mutation, especially in parasitic lineages (Krause, 2008). When ReFernment was tested against plastomes with high levels of RNA editing, confirmed with cDNA data (AB086179 and AY178864.1), every nonsense mutation was correctly annotated, and there were no false positive annotations. A major limitation of ReFernment is that the annotations it produces are only as good as the annotations it is provided. If a gene annotation is frameshifted, if a pseudogene is annotated as a coding sequence, if there are assembly errors, or if an annotation has the incorrect start and stop sites, ReFernment might interpret this as RNA editing, rather than an error. In other words, ReFernment is not a substitute for manually checking gene annotations, nor is ReFernment a fix for sloppy annotation. In an attempt to mitigate these problems, if there are more than five detected internal stops in a gene, ReFernment will produce an error suggesting that the user manually check that gene. There are cases where genes have more than five RNA‐edited internal stops, but these are relatively rare, so users should use best judgement.

The utility of ReFernment is simple: it saves users time in the final stages of annotation. Manually accounting for RNA edits generally takes hours for a typical fern or hornwort plastid genome, but with ReFernment, this process takes less than a minute. There are currently efforts to publish some 1000 additional fern plastomes in the coming years, and hopefully similar efforts are underway for hornworts, meaning many thousands of hours can be saved by the implementation of this simple program. ReFernment not only saves the researcher time, but also provides consistent methodology for the annotation of RNA editing. In many cases, RNA editing sites are not annotated in plastid sequences and only conceptual translations are provided. This not only results in confusion in how to annotate such sites consistently, but also makes it difficult for researchers interested in the evolution of such sites to readily identify them.

## CONCLUSIONS

ReFernment offers easy and rapid annotation of RNA‐edited sites and automatic conceptual translation of amino acid sequences, streamlining the process of GenBank submission and saving the user valuable time.

## Data Availability

The Refernment software is published under the GNU General Public License (version 3); the software and related documentation are available for free download at GitHub (https://github.com/TARobison/ReFernment).
